# Predicting mammalian species at risk of being infected by SARS-CoV-2 from an ACE2 perspective

**DOI:** 10.1038/s41598-020-80573-x

**Published:** 2021-01-18

**Authors:** Yulong Wei, Parisa Aris, Heba Farookhi, Xuhua Xia

**Affiliations:** 1 Department of Biology, University of Ottawa, 30 Marie Curie, Station A, P.O. Box 450, Ottawa, ON K1N 6N5 Canada; 2 Ottawa Institute of Systems Biology, Ottawa, ON K1H 8M5 Canada

**Keywords:** Evolution, Genetics, Molecular biology

## Abstract

SARS-CoV-2 can transmit efficiently in humans, but it is less clear which other mammals are at risk of being infected. SARS-CoV-2 encodes a Spike (S) protein that binds to human ACE2 receptor to mediate cell entry. A species with a human-like ACE2 receptor could therefore be at risk of being infected by SARS-CoV-2. We compared between 132 mammalian *ACE2* genes and between 17 coronavirus S proteins. We showed that while global similarities reflected by whole *ACE2* gene alignments are poor predictors of high-risk mammals, local similarities at key S protein-binding sites highlight several high-risk mammals that share good *ACE2* homology with human. Bats are likely reservoirs of SARS-CoV-2, but there are other high-risk mammals that share better *ACE2* homologies with human. Both SARS-CoV-2 and SARS-CoV are closely related to bat coronavirus. Yet, among host-specific coronaviruses infecting high-risk mammals, key ACE2-binding sites on S proteins share highest similarities between SARS-CoV-2 and Pangolin-CoV and between SARS-CoV and Civet-CoV. These results suggest that direct coronavirus transmission from bat to human is unlikely, and that rapid adaptation of a bat SARS-like coronavirus in different high-risk intermediate hosts could have allowed it to acquire distinct high binding potential between S protein and human-like ACE2 receptors.

## Introduction

The *Betacoronavirus* SARS-CoV-2 poses a serious global health emergency. Since its emergence in Wuhan city, Hubei province of China in December 2019, the viral outbreak has resulted in over 20 million confirmed cases of COVID-19 worldwide (https://www.who.int/emergencies/diseases/novel-coronavirus-2019, last accessed August 25, 2020). While it is evident that SARS-CoV-2 can transmit efficiently from person to person, it is less clear which other mammalian species are at high risk of being infected. The answer to this question is important to (1) improve our ability to predict and control future pandemics, and (2) manage and protect wildlife and domesticated animals.

### Mammals at high risk of SARS-CoV-2 infection should have human-like ACE2 receptors at key S protein-binding sites

Both SARS-CoV and SARS-CoV-2 genomes encode a Spike (S) protein that binds to human Angiotensin-converting enzyme 2 (ACE2) receptor to mediate viral entry into the host cell^1–6^. Mechanistically, two S protein domains are involved during coronavirus infection in mammalian cells that express ACE2: the S1 domain interacts with the ACE2 receptor^7,8^, and the S2 domain undergoes structural rearrangements to mediate membrane fusion^4^. Interacting with the S1 domain, the S protein-binding sites on ACE2 receptor are primarily located in the α-helix 1 and β-sheet 5 domains^4^. The efficacy of the interaction between the S protein and the ACE2 receptor is a good predictor of the severity of coronavirus infection^4,9,10^. For example, a potential source of the SARS-CoV outbreak was the masked palm civets^11,12^. Palm civet ACE2 receptors bind efficiently to S proteins of Civet-CoV strain SZ3 isolated from infected palm civets, to S proteins of SARS-CoV strain TOR2 that caused the severe 2002–2003 outbreak, and to S proteins of SARS-CoV strain GD that caused the mild 2003–2004 outbreak^11–14^. Whereas in human cells, ACE2 receptors bind efficiently to S proteins of the severe SARS-CoV strain TOR2 but do not bind efficiently to S proteins of Civet-CoV strain SZ3 or of the less severe SARS-CoV strain GD^4^. Indeed, the binding potential between viral S protein and host ACE2 receptor is a key determinant of viral infectivity.

The binding potential between viral S protein and host ACE2 receptor is attributed to several key binding sites. Differences at key S protein-binding sites between mammalian ACE2 receptors explained why SARS-CoV efficiently infected humans and palm civets but not rats, and introducing point mutations in the *ACE2* gene variably affected the binding potential between ACE2 receptor and SARS-CoV S protein in these species^4^. For example, experimentally mutating rat His353 into human Lys353 turned the rat ACE2 receptor from one that poorly binds S protein into one that is efficient for binding, introducing amino acid residues 82–84 from human *ACE2* into rat *ACE2* also led to an increase in S protein-binding potential, but replacing human *ACE2* Met82 to rat *ACE2* Asn82 partially inhibited S protein-binding. Furthermore, mutating human *AEC2* at Lys31, Tyr41, Asp355, and Arg357 also interfered with S protein-binding. On the contrary, changes to other human *ACE2* sites such as Gly354 (corresponding to Asp354 in palm civet) and amino acid residues 90–93 that potentially interact with S protein residue 479 did not affect the efficacy of S protein-binding^10^. In brief, gene mutation experiments^3,4^ showed that the conservation of Lys31 and Tyr41 on α-helix 1, residues 82–84 in the vicinity of α-helix 3, and residues 353–357 on β-sheet 5 in human ACE2 receptor are crucial for SARS-CoV infectivity because their replacements weakened the binding potential between SARS-CoV S protein and ACE2 receptor.

**Figure 1:**
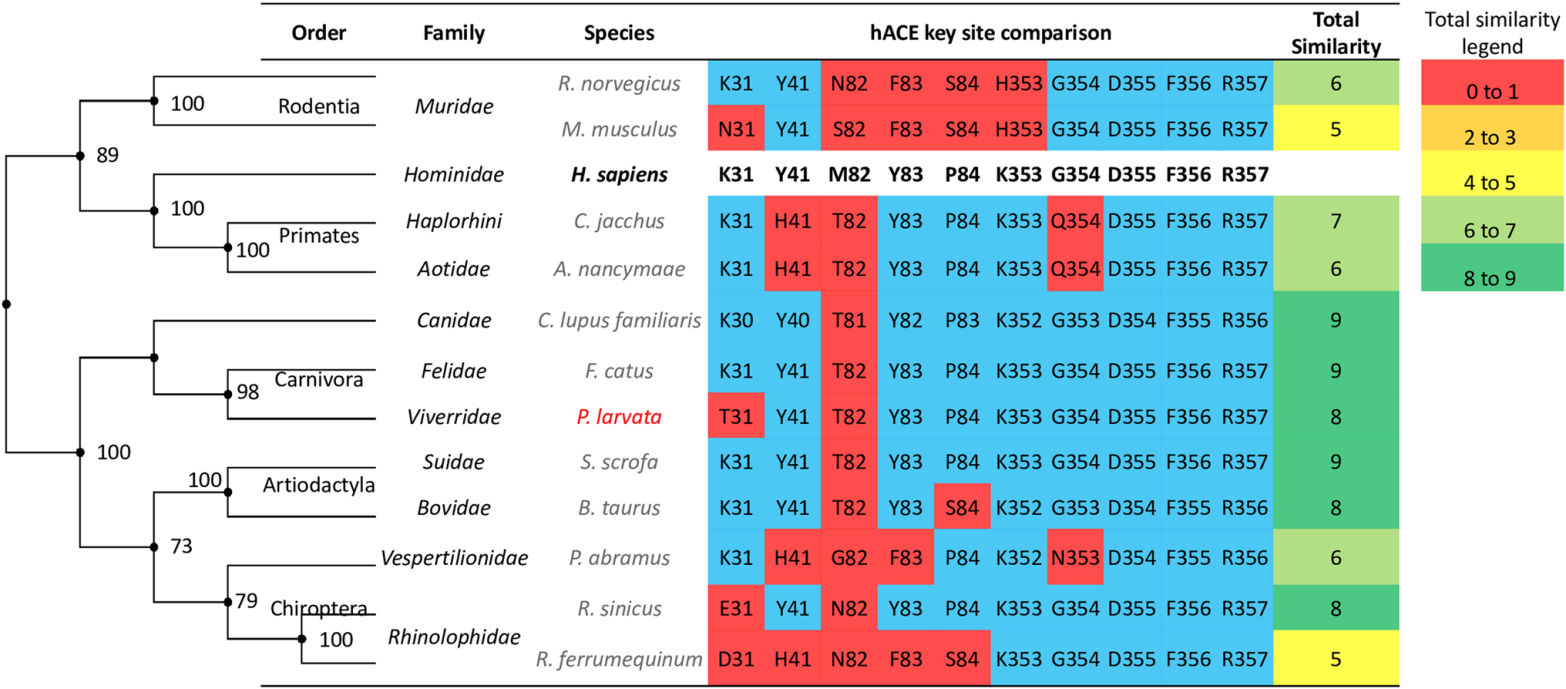
A sample comparison between mammalian *ACE2* genes. Global similarities represented by whole *ACE2* gene comparisons (the phylogenetic tree on the left) poorly predict mammals at high risk of being infected by SARS-CoV; whereas local similarities at key human *ACE2* sites (the table on the right) show that species in the Carnivora, Artiodactyla and Chiroptera orders are at high risk of SARS-CoV infection. The tips of the phylogenetic tree are aligned with the corresponding species listed in the table. Species in red is the masked palm civet, a possible intermediate host of SARS-CoV. Species in black bold is human, and all other species are in grey. Amino acids highlighted in blue and red are matching and mis-matching sites, respectively, between mammalian *ACE2* and human *ACE2* at key SARS-CoV S protein binding sites. Total similarity in the table is calculated as the sum of matching amino acids between mammalian and human *ACE2* at key binding sites. A comparison of key *ACE2* sites involved in SARS-CoV binding between human and 131 mammals is shown in Supplementary Figure S3.

To showcase the importance of contrasting between mammalian *ACE2* genes at key S protein-binding sites, Fig. 1 compares *ACE2* genes in a sample of mammalian species in two ways: the global similarities among whole *ACE2* gene alignments as reflected by the mammalian phylogenetic relationships on the left, and the local similarities at key *ACE2* sites that are involved in SARS-CoV S protein-binding^3,4^ in the table on the right. Figure 1 shows that human *ACE2* shares higher global similarity with *ACE2* of species from Primates and Rodentia orders than with *ACE2* of species from Carnivora, Artiodactyla, and Chiroptera orders. This emphasizes that a phylogenetic relationship based on whole *ACE2* gene comparisons is not a good predictor of mammals at high risk of being infected by SARS-CoV for the following three reasons. First, it does not identify the *Rhinolophus* bats as a potential reservoir for the progenitor virus of SARS-CoV as previously postulated^15^. Second, it does not reveal masked palm civets as a potential intermediate host^11,12^. Third, it misidentifies the rat as a high-risk mammal while experimental evidence suggests SARS-CoV poorly infects the rat^4^. In contrast, comparisons of *ACE2* genes at key SARS-CoV S protein-binding sites show that species in the Carnivora, Artiodactyla, and Chiroptera orders share highest local similarities with human. These high-risk mammals include the *Rhinolophus* bat and masked palm civets, even though they share less global *ACE2* similarity with human than Primates and Rodentia species. Hence, comparing key binding sites on *ACE2* may provide insights into the host range of viral infection.

The above observations allow for the prediction that high-risk mammals of the newly emerging SARS-CoV-2 should have human-like ACE2 receptors at key SARS-CoV-2 S protein-binding sites. Current research on SARS-COV-2 infection in animals is limited. Nonetheless, SARS-CoV-2 infection has been detected in the cat^16^, dog^17^, mink^18^, and tiger^19^. In addition, ferret^20,21^, macaque^22,23^, and grivet^24^ could be experimentally infected by SARS-CoV-2. We therefore expect the *ACE2* genes of these known high-risk mammals to bear a high resemblance to human *ACE2* at key S protein-binding sites. Moreover, two Rodentia species have been experimentally infected by SARS-CoV-2: the golden Syrian hamster (*Mesocricetus auratus*) belonging to the *Cricetidae* family, and the house mouse (*Mus musculus*) belonging to the *Muridae* family. While hamsters could be consistently infected by SARS-CoV-2^25^, wild-type mice were not susceptible to SARS-CoV-2 infection^26^. It follows that these two Rodentia species should have distinct differences at the ACE2 receptor, and we expect key *ACE2* sites in human to be well conserved by hamster *ACE2* but not by mouse *ACE2*. Sequence comparisons between human ACE2 receptor and mammalian ACE2 receptors at key S protein-binding sites may provide crucial insights into the identification of mammals at high risk of SARS-CoV-2 infection.

Human ACE2 receptors bind to S proteins on SARS-CoV and on SARS-CoV-2 with overall structural similarity; this is likely constrained by the structure of ACE2^2^. However, the S protein-coding genes are notably different between SARS-CoV and SARS-CoV-2 with about 75% homology^6,27^. This dissimilarity may contribute to a difference in viral infectivity and host range between the two SARS viruses. Indeed, based on two recent X-ray crystallography experiments^2,5^, different key residues on human ACE2 receptor are structurally involved in binding to S proteins of SARS-CoV-2 and SARS-CoV. Table 1 summarizes all experimentally identified site-specific interactions at the ACE2-S protein interface^2,5^. Four key human ACE2 sites bind to S protein of SARS-CoV-2 but they do not interact with S protein of SARS-CoV: ACE2 Ser19 forms a hydrogen bond with S protein Ala475, ACE2 Asp30 forms a hydrogen bond with S protein at Lys417, ACE2 Glu35 forms a hydrogen bond with S protein Gln493, and ACE2 Arg393 forms a hydrogen bond with S protein Tyr505. Furthermore, there are 11 other key human ACE2 sites (Gln24, Lys31, His34, Glu37, Asp38, Tyr41, Gln42, Leu79, Met82, Tyr83, and Lys353) that form either hydrogen bonds or salt bridges with S proteins of both SARS-CoV-2 and SARS-CoV, but they interact with different S protein residues. For example, ACE2 Gln24 forms a hydrogen bond with SARS-CoV-2 S protein at Leu472 but with SARS-CoV S protein at Asn473, and ACE2 Tyr41 forms a hydrogen bond with SARS-CoV-2 S protein at Thr500 and Asn501 but with SARS-CoV S protein at Thr486 and Thr487. The extended data in Lan, et al.^2^ also highlights ACE2 sites Thr27, Phe28, Asn330, Gly354 and Asp355 to be in proximity with SARS-CoV-2 S protein. However, these residues were excluded from analysis because chemical bond evidence was not shown at these sites. In brief, we analyzed all key interacting residues (listed in Table 1) whose structures were shown and whose chemical bond evidences were reported, by one or both X-ray crystallography experiments^2,5^ with structure resolution cut-off < 3 Å and interface distance cut-off < 5 Å. Nonetheless, it is worth noting that future gene mutation studies should be performed to verify whether all 15 key ACE2 sites are essential for SARS-CoV-2 infection.

**Table 1:** Contacting sites at the interface of ACE2-S protein of SARS-CoV-2 (column 1, 2) and of SARS-CoV (column 3, 4).

Human ACE2	SARS-CoV-2	Human ACE2	SARS-CoV
*S19*^a^	A475^a^	**Q24**^b^	N473^b^
**Q24**^b^	N487^b^	**K31**^a,b^	Y442^a,b^
**Q24**^b^**, M82**^a,b^**, L79**^a,b^**, Y83**^a,b^	F486^a,b^	**H34**^b^	L443^b^, N479^b^
*D30*^b^	K417^b^	**E37**^b^	Y491^a,b^
**K31**^a,b^	L455^a,b^, Q493^b^	**D38**^a,b^	Y436^b^, Y484^b^
**H34**^b^**,** *E35*^a,b^	F456^b^, Q493^a,b^	**Y41**^b^	Y484^b^, T486^b^, T487^b^
**E37**^b^, *R393*^b^	Y505^b^	**Q42**^b^	Y436^b^, Y484^b^
**D38**^a,b^	Y449^b^, Q498^b^	**L79**^b^**, M82**^a,b^	L472^a,b^,
**Y41**^b^	Q498^b^, T500^b^, N501^b^	**Y83**^b^	Y475^b^, N473^b^
**Q42**^b^	G446^b^, Y449^b^, Q498^b^	*Q325*^b^*, E329*^b^	R426^b^
**Y83**^b^	Y489^b^, N487^b^	*N330*^b^	T486^b^
**K353**^a,b^	N501^a^, G502^b^	**K353**^a,b^	T487^a^, G488^b^

Data are retrieved from X-ray crystallography experiments by Shang et al.^5^ and Lan et al.^2^. Each row specifies the site-specific contacts formed between human ACE2 and S protein (e.g., first row: ACE2 S19 binds SARS-CoV-2 S protein A475). In italics are key ACE2 sites that distinctly interact with SARS-CoV-2 or SARS-CoV S protein sites. In bold are ACE2 sites that interact with both SARS-CoV-2 and SARS-CoV S proteins at different ACE2-binding sites. The structural and chemical bond evidence for all listed interactions were described in Shang et al.^5^ and Lan et al.^2^

^a^Interacting residues retrieved from Shang et al.^5^

^b^Interacting residues retrieved from Lan et al.^2^

### Species-specific coronaviruses infecting high-risk mammals may express SARS-like S proteins at key ACE2-binding sites

Next, we wish to know which mammalian-specific coronaviruses are similar to SARS-CoV-2 and to SARS-CoV in infectivity. We discussed above that a high-risk mammal that is potentially capable of carrying SARS viruses should express a human-like ACE2 receptor, it follows that its host-specific coronavirus may be SARS-like in infectivity. Just as mammals at high risk of SARS-CoV-2 infection should have human-like ACE2 receptors at key S protein-binding sites**,** we expect SARS-like mammalian coronaviruses to express SARS-like S proteins at key ACE2-binding sites. Table 1 details 15 key SARS-CoV-2 S protein sites and 13 key SARS-CoV S protein sites that were deemed as essential in human ACE2-binding^2,5^. Determining similarities at key ACE2-binding sites among S proteins may help identify mammalian coronaviruses that have SARS-like infectivity and shed light on the zoonosis of SARS-CoV-2.

To expand on the above point, many recent studies point to the bat *Betacoronavirus* RaTG13 as a close relative of SARS-CoV-2^5,28,29^. SARS-CoV-2 S protein contains a unique Gly482, Val483, Glu484, and Gly485 four-residue motif in the binding ridge that may facilitate contact with human ACE2 N-terminal helix. Indeed, bat RaTG13 also contains a similar four-residue motif^5^. Moreover, residues Leu455 and Asn501 on SARS-CoV-2 S protein are conserved by RaTG13 S protein^5^; these sites contribute favorably to human ACE2-binding because their mutations reduced binding potential. These observations may explain why RaTG13 could use human ACE2 as its receptor^5^. Nonetheless, many residues in RaTG13 S protein are not fine-tuned for binding with human ACE2^2,5^. Changing bat RaTG13 S protein at residues Lys486 and Tyr493 to SARS-CoV-2 S protein residues Phe486 and Gln493, respectively, enhanced human ACE2 recognition^5^. Besides bat RaTG13, the pangolin *Betacoronavirus* Pangolin-CoV also shares high sequence similarity with SARS-CoV-2^28^ and contains the 482–485 four-residue motif in its S protein^5^. Furthermore, key ACE2-binding sites such as Leu455, Phe486, Gln493, and Asn501 are conserved between SARS-CoV-2 and Pangolin-CoV S proteins. Indeed, both bats and pangolins have been proposed to be potential intermediate hosts for SARS-CoV-2, and host-specific coronaviruses of these two mammals bear a resemblance to SARS-CoV-2 at key S protein sites^30^.

Our investigation considered 132 *ACE2* genes from mammals across 19 orders and 17 mammalian-specific coronaviruses infecting high-risk species. Key *ACE2* sites are strongly conserved among Primates. Among other mammals, key binding sites on human *ACE2* are conserved by selected species of the Chiroptera, Artiodactyla, Rodentia, Carnivora, Perissodactyla, Pholidota, Lagomorpha, Proboscidea, and Sirenia orders. Among these high-risk mammals, 12 species are known to be infected by host-specific coronaviruses. We found that key S protein sites in SARS-CoV are most conserved by Civet-CoV, whereas key S protein sites in SARS-CoV-2 are most conserved by Pangolin-CoV. Both SARS viruses also share several key S protein sites with bat RaTG13 but not with other mammalian-specific coronaviruses. Together, our results reinforce the current hypothesis that the progenitor of both SARS-CoV-2 and SARS-CoV are likely of bat origin. The palm civet and pangolin may have served as distinct intermediate hosts that facilitated the adaptation of SARS viruses to bind human ACE2 receptors prior to zoonosis because both mammals express human-like ACE2 receptors at key S protein-binding sites and their host-specific coronaviruses encode S proteins that share highest similarities with S proteins of SARS viruses at key ACE2-binding sites.

## Results

### Key binding sites on the human ACE2 receptor are most conserved by primates species and variably conserved in selected species belonging to eight other mammalian orders

We investigated 132 species belongs to 19 mammalian orders with available *ACE2* gene records. Among Primates, key sites on human *ACE2* are perfectly conserved by species belonging to the *Hominidae* and *Cercopithecoidae* families and highly conserved by other species (Supplementary Fig. S1). Among the other 18 orders, key *ACE2* sites are highly conserved in selected species belonging to eight orders: Artiodactyla, Chiroptera, Carnivora, Rodentia, Lagomorpha, Perissodactyla, Proboscidea, and Sirenia (Supplementary Fig. S1). More specifically, in the Artiodactyla order, species belonging to the *Bovidae*, *Monodontidae*, *Phocoenidae*, and *Physeteridae* families share very high similarities with human at key *ACE2* sites. In the Chiroptera order, species belonging to the *Pteropodidae* and *Rhinolophidae* share high similarities with human at key *ACE2* sites. In the Carnivora order, species belonging to the *Canidae*, *Felidae*, and *Ursidae* families share high similarities with human at key *ACE2* sites. In the Rodentia order, all species (e.g., belonging to *Cricetidae*, *Sciuridae*) share high similarities with human at key *ACE2* sites except species in the *Muridae* family. In the other four orders, the specific species that share high similarities with human at key *ACE2* sites are *Ochotona princeps* and *Oryctolagus cuniculus* (order Lagomorpha), *Ceratotherium simum* (order Perissodactyla), *Loxodonta Africana* (order Proboscidea), and *Trichechus manatus latirostris* (order Sirenia). Similar to the SARS-CoV example displayed in Fig. 1, while local *ACE2* site comparisons suggest that select species among nine orders could be at high risk to SARS-CoV-2 infection (Supplementary Fig. S1), global similarities among whole *ACE2* gene alignments (Supplementary Fig. S2) grouped 132 species by order and did not point out any species as high-risk.

**Figure 2:**
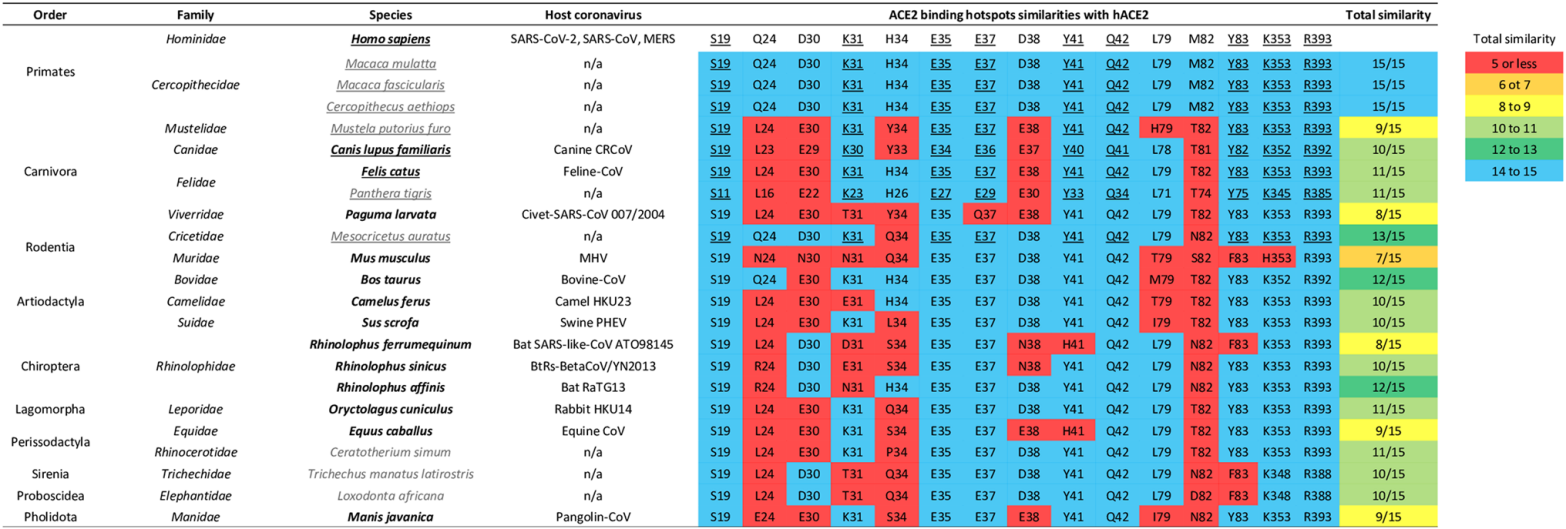
Comparisons at key *ACE2* sites between human and a sample of mammals belong to ten orders. Species that have been experimentally identified as susceptible to SARS-CoV-2 infection are underlined. Species that can be infected by their own host-specific coronaviruses are in black bold, and other species are in grey. Highlighted blue and red are matching and mis-matching amino acid residues, respectively, between mammalian *ACE2* and human *ACE2* at key SARS-CoV-2 S protein binding sites. Underlined amino acids are conserved among species known to be infected by SARS-CoV-2. Total similarity designates the total number of matching amino acid residues with respect to human *ACE2*. The total similarity score for mammalian *ACE2* in blue highlights perfect site similarities (14–15 matching sites), in green highlights high similarity (12–13 matching sites), in light green highlights medium–high similarity (10–11 matching sites), in yellow highlights medium similarity (8–9 matching sites), in orange highlights medium–low similarity (6–7 matching sites), and in red highlights low similarity (5 or less matching sites). A comparison of key *ACE2* sites involved in SARS-CoV-2 binding between human and 131 mammals is shown in Supplementary Figure S1.

Figure 2 showcases key *ACE2* site comparisons among a sample of mammals. These species were selected based on the following three criteria: (1) they all belong to the nine orders that contain high-risk mammals (Primates, Artiodactyla, Chiroptera, Carnivora, Rodentia, Lagomorpha, Perissodactyla, Proboscidea, and Sirenia), (2) all species that have been experimentally identified as susceptible to SARS-CoV-2 infection were selected (underlined), and (3) some species were selected because they are known to be infected by their own host-specific coronaviruses (in bold). In addition, the pangolin (*Manis javanica*) belonging to the Pholidota order shares medium similarity with human at key *ACE2* sites; the species was added to Fig. 2 because it has been proposed as a possible intermediate host of SARS-CoV-2^30^.

All mammals currently known to be susceptible to SARS-CoV-2 infection share similarity with human at key *ACE2* sites (Fig. 2). These include cat (*Felis catus*)^16^, dog (*Canis lupus familiaris*)^17^, and tiger (*Panthera tigris*)^19^ where SARS-CoV-2 infections were detected, and ferret (*Mustela putorius furo*)^20,21^, macaque (*Macaca mulatta*^22^ and *Macaca fascicularis*^23^), and grivet (*Cercopithecus aethiops*)^24^ that were experimentally infected by SARS-CoV-2. One additional mammal that is known to be infected by SARS-CoV-2 is the mink (*Neovison vison*)^18^, a relative of the ferret. However, the mink was not included in Fig. 2 because there were no *ACE2* gene records for this species in the NCBI gene database (last accessed April 25, 2020). Indeed, the *ACE2* genes in all these species share high similarity with human *ACE2* at key binding sites, except for ferret, which shares medium similarity. Wild-type mouse (*Mus musculus*) is not susceptible to SARS-CoV-2 infection^26^, and expectedly, the *ACE2* gene in mouse shares poor similarity with human *ACE2* at key binding sites (Fig. 2).

Among mammals that are known to be infected by SARS-CoV-2, nine key human *ACE2* sites (Ser19, Lys31, Glu35, Glu37, Tyr41, Gln42, Tyr83, Lys353 and Arg393) are conserved by their mammalian *ACE2* genes, but the other six key sites (Gln24, Asp30, His34, Asp38, Leu79, and Met82) are not well conserved (Fig. 2). One may reason that only these nine conserved ACE2 sites could be important in S protein-binding. Nonetheless, mis-match amino acids at the six non-conserved sites all share similar physiochemical properties with the human *ACE2* residue (the replacements resulted in small Graham’s distance D^31^, which implies similar composition, polarity, and molecular volume between the two amino acids). Aspartic acid at sites 30 and 38 are replaced by Glutamic acid (Grantham’s distance D = 45), Glutamine at site 24 is replaced by Lysine (D = 53), Histidine at site 34 is replaced by Tyrosine (D = 83), and Methionine at site 82 is replaced by Threonine (D = 81). Future *ACE2* gene mutation studies may be required to better elucidate which key *ACE2* sites among the 15 contacting residues are essential for SARS-CoV-2 infection.

### Key ACE2-binding sites on SARS-CoV-2 and SARS-CoV S proteins are distinctly conserved by mammalian coronaviruses

Above results highlighted several mammals at high risk of SARS-CoV-2 infection. Figure 2 also highlights 12 species-specific coronaviruses that infect high-risk mammals (belonging to the Artiodactyla, Canivora, Chiroptera, Perissodactyla, Pholidota, and Lagomorpha orders) and one MHV coronavirus that infects the low-risk mice, but mammals of the Proboscidea and Sirenia orders do not have records of species-specific coronaviruses. Some of these coronaviruses may have SARS-like infectivity because they infect mammals having human-like ACE2 receptors. We thus investigated whether key S protein sites in SARS-CoV-2 and SARS-CoV are conserved by mammalian-specific coronaviruses.

**Figure 3:**
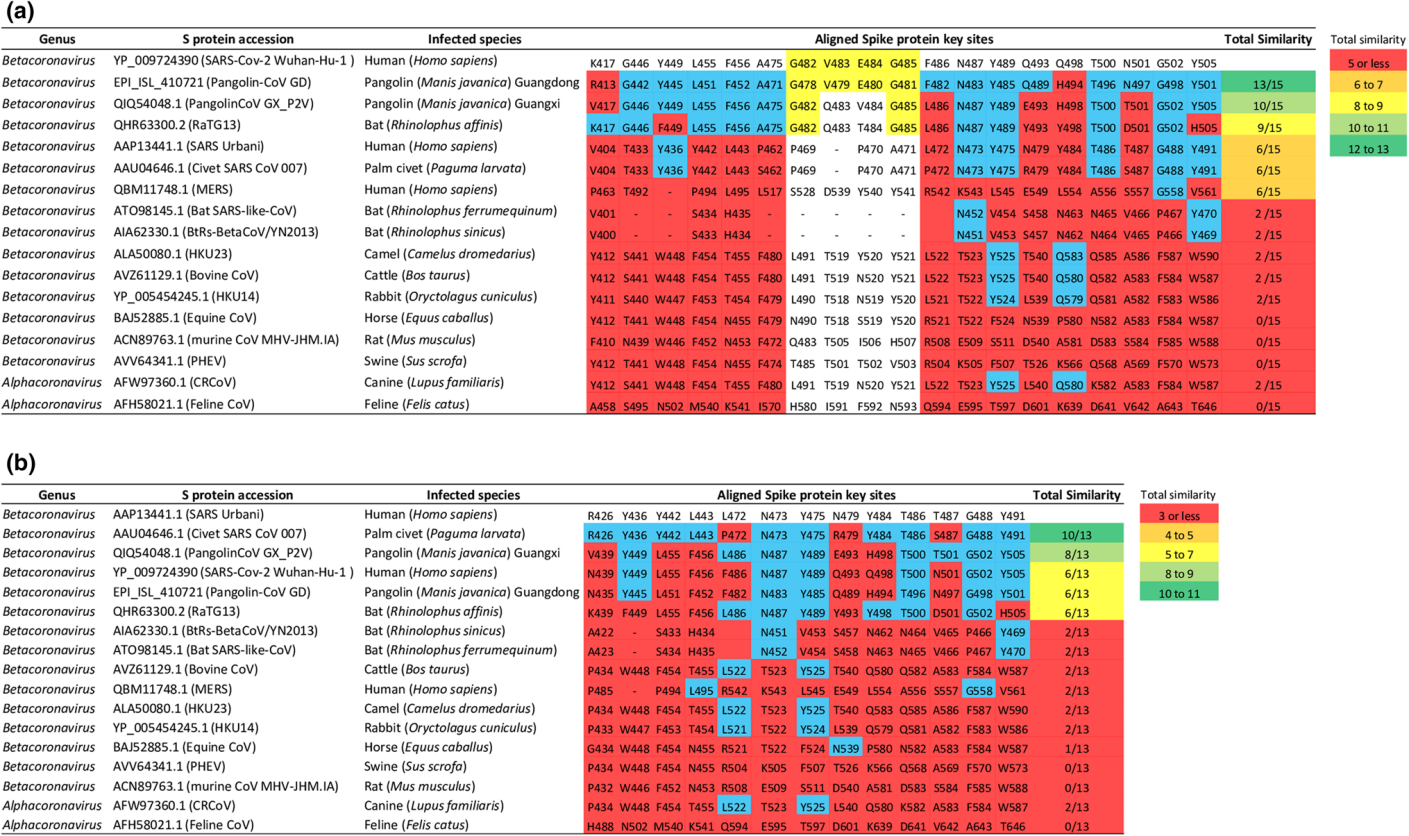
Amino acid comparisons at key S protein sites among 17 mammalian-specific coronaviruses. (**a**) Displays the conservation of key ACE2-binding sites on SARS-CoV-2 S protein by 16 mammalian-specific S proteins. (**b**) Displays the conservation of key ACE2-binding sites on SARS-CoV S protein by 16 mammalian-specific S proteins. Highlighted blue and red are matching and mis-matching amino acid residues, respectively, between mammalian coronaviruses and the two human SARS coronaviruses at key S protein sites. Highlighted yellow is the 482–485 GVEG motif found in SARS-CoV-2. Total similarity designates the total number of matching amino acid residues with respect to SARS-CoV-2 or SARS-CoV, and scores in green highlights high similarity, in light green highlights medium–high similarity, in yellow highlights medium similarity, in orange highlights medium–low similarity, and in red highlights low similarity.

Figure 3 shows distinct differences in the conservation of key SARS-CoV-2 and SARS-CoV S protein sites by mammalian-specific coronaviruses. Between the two SARS viruses, key S protein sites of SARS-CoV-2 are weakly conserved by SARS-CoV (Fig. 3a) and vice versa (Fig. 3b). In addition, key S protein sites of both SARS viruses share a medium degree of similarity with S protein sites of bat coronavirus RaTG13 isolated from *Rhinolophus affinis*, but they share poor similarities with S protein sites of two other bat coronaviruses isolated from *Rhinolopus sinicus* and *Rhinolopus ferrumequinum*. Importantly, key S protein sites of SARS-CoV-2 share highest similarity with S protein sites of Pangolin-CoV strain Guangdong (GD) that was sequenced with high coverage, but share lower similarity with S protein sites of Pangolin-CoV strain Guangxi (GX) that was flagged as poorly sequenced by GISAID (Fig. 3a). Indeed, the unique 482–485 domain in SARS-CoV-2 (highlighted yellow, Fig. 3a) is perfectly conserved by Pangolin-CoV GD, partially conserved by bat RaTG13 and Pangolin-CoV GX, and not conserved by any other coronaviruses surveyed. In contrast, key S protein sites of SARS-CoV share highest similarity with S protein sites of Civet-CoV but share medium similarity with S protein sites of Pangolin-CoVs (Fig. 3b). As for other mammalian-specific coronaviruses, including the human MERS-CoV with a presumed camel origin, their S proteins share little similarities with SARS-CoV-2 and SARS-CoV at key sites. Together, these findings imply that the bat coronavirus RaTG13 is SARS-like in infectivity, but SARS-CoV-2 more closely resembles Pangolin-CoV and SARS-CoV more closely resembles Civet-CoV.

**Figure 4:**
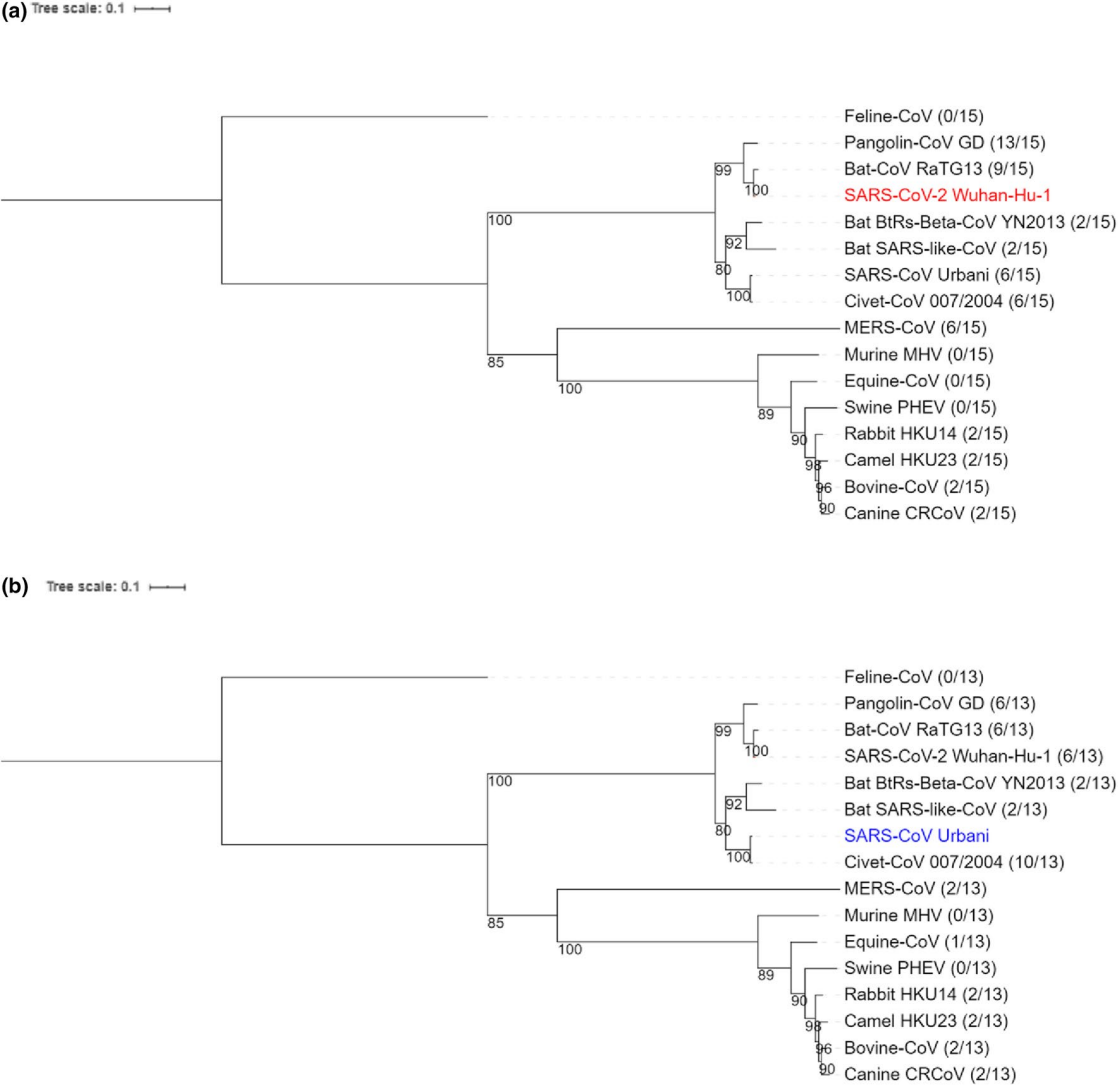
Phylogenetic reconstruction of 16 MAFFT G-INS-i aligned coronavirus S protein-coding genes. Infected mammalian species belong to the Artiodactyla, Canivora, Chiroptera, Lagomorpha, Perissodactyla, Primates, Pholidota and Rodentia orders. The phylogenetic tree is constructed using the maximum-likelihood-based PHYML approach, with best model = WAG + G + I + F, and Bootstrap = 100. Pangolin-CoV GX strain was excluded in favor of Pangolin-CoV GD strain having better sequence coverage. The ratio appended to each coronavirus indicates the total number of amino acid matches when coronavirus S protein gene was compared to (**a**) SARS-CoV-2 S protein gene (in red) at its 15 key ACE2-binding sites and to (**b**) SARS-CoV S protein gene (in blue) at its 13 key ACE-2 binding sites.

Global similarities among whole S protein-coding amino acid sequences (Fig. 4) also showed that SARS viruses are distinctly closely related to mammalian coronaviruses: SARS-CoV-2 closely relates to Pangolin-CoV and bat RaTG13 (Fig. 4a), and SARS-CoV closely relates to Civet-CoV (Fig. 4b). However, while SARS-CoV-2 is more similar to bat RaTG13 than to Pangolin-CoV in terms of global S protein similarity (Fig. 4a), key S protein sites are more similar between SARS-CoV-2 and Pangolin-CoV (Fig. 3a). The notion that comparing local but not global S protein similarities is crucial in determining SARS-like coronavirus S proteins was therefore consistent with the notion that comparing local but not global *ACE2* similarities is crucial in determining mammals at high risk of infection by SARS viruses (Figs. 1, 2).

## Discussion

SARS-CoV-2 can transmit efficiently in humans, but it is less clear which other mammals are at risk of being infected. We performed comparative gene analyses to trace differences at the *ACE2* gene of 132 mammalian species belonging to 19 orders. Similarities in mammalian *ACE2* genes were measured in two ways, one was global similarities reflected by the phylogenetic relationship from whole *ACE2* sequence alignments, and the other was local comparisons at key human *ACE2* sites. While global similarities (Supplementary Fig. S2) were not good predictors of mammals at high-risk of being infected by SARS-CoV-2, local similarities highlighted several high-risk mammals belonging to nine out of 19 orders surveyed (Fig. 2, Supplementary Fig. S1: Primates, Artiodactyla, Canivora, Chiroptera, Lagomorpha, Perissodactyla, Proboscidea, Rodentia and Sirenia).

Species currently known to be susceptible to SARS-CoV-2 infection are indeed high-risk mammals that share high similarities with human at key *ACE2* sites (Fig. 2). For example, while golden Syrian hamster could be consistently infected by SARS-CoV-2^25^ and its *ACE2* gene shares high local similarity with human *ACE2* gene, wild-type mouse could not be infected by SARS-CoV-2^26^ and its *ACE2* gene expectedly shares poor local similarity with human *ACE2* gene. However, these differences between the two Rodentia species could not be distinguished from global *ACE2* sequence similarities (Supplementary Fig. S2). Among other susceptible mammals (Fig. 2), confirmed cases of SARS-CoV-2 infection have been reported for domesticated cats and dogs across several U.S. states (https://www.aphis.usda.gov/aphis/ourfocus/animalhealth/sa_one_health/sars-cov-2-animals-us, last accessed August 25, 2020). These findings may prompt future investigations to perform SARS-CoV-2 screening in other high-risk mammals, especially other domesticated animals living in proximity with humans such as pigs and cattle. Indeed, the pig was previously predicted to be susceptible to SARS-CoV-2 infection based on computational models of ACE2 structures^32^, but SARS-CoV-2 infection in pig has yet to be detected^21,33^.

We also analyzed which host-specific coronaviruses infecting high-risk mammals could be SARS-like in infectivity. To this end, we measured global similarities at whole S protein-coding genes and local similarities at key S protein sites among 17 coronaviruses infecting human, 12 high-risk mammals, and the low-risk mouse. We showed that key S protein sites of the two SARS viruses are modestly conserved by one bat coronavirus RaTG13. More importantly, key S protein sites in SARS-CoV and SARS-CoV-2 are distinctly most conserved by Civet-CoV and Pangolin-CoV GD, respectively (Fig. 3). Indeed, a recent study^28^ had also found that SARS-CoV-2 is more similar to Pangolin-CoV GD than to bat RaTG13 or to SARS-CoV at the S1 binding domain of the S protein. However, based on global similarities, the S protein of SARS-CoV-2 is more closely related to bat RaTG13 than it is to Pangolin-CoV (Fig. 4), similar to what others have shown^2,34^. Hence, similarities in coronavirus infectivity can be better determined by local than global S protein sequence alignments. Nevertheless, both Figs. 3 and 4 suggest that the two SARS viruses are evolutionarily distinct in terms of infectivity.

Our results corroborate the current hypothesis on the origin and evolution of SARS viruses. The progenitors of SARS-CoV-2 and SARS-CoV are likely to have a bat coronavirus origin^1,35^, and the bat serves as a potential reservoir for these viruses. However, transmissions of SARS viruses from bats to humans are unlikely, and the viruses may had required adaptation in distinct intermediate hosts, namely the palm civet for SARS-CoV^4,11^ and the pangolin for SARS-CoV-2^30,34,36^. Indeed, the *ACE2* genes in both palm civet and pangolin share similarities with human *ACE2* gene at key S protein-binding sites. Additionally, key S protein sites are most conserved between Civet-CoV and SARS-CoV, and between Pangolin-CoV and SARS-CoV-2. It is plausible that, prior to zoonotic transmission, rapid evolution of progenitor SARS virus in distinct intermediate hosts allowed it to adapt high binding potential between viral S protein and human-like ACE2 receptor and led to differences between SARS-CoV-2 and SARS-CoV at the S protein.

## Methods

### Retrieving and processing 132 mammalian *ACE2* genes and 17 coronavirus S protein-coding genes

The nucleotide sequences of 266 *ACE2* gene variants from 132 mammalian species were retrieved from the National Center for Biotechnology Information (NCBI) Nucleotide Database (https://www.ncbi.nlm.nih.gov/). The NCBI Nucleotide Database was queried for records containing “ACE2” as gene name and “Mammalia” as taxonomic class, excluding whole-genome and chromosome-wide results. Next, each record was searched for /product = “angiotensin-converting enzyme 2”, and all others were removed. For each *ACE2* gene entry, only the coding DNA sequence region was extracted in FASTA format. The coding DNA sequences were translated from nucleotides into amino acids using DAMBE7^37^ and verified with annotated amino acid sequences from NCBI GenBank files. Then, for sequence files, gene IDs were renamed as follow: ACE2_NCBI gene accession ID_Species name (Supplementary File S1). Similarly, the amino acid sequences of S proteins encoded by 17 host-specific coronaviruses infecting 12 mammalian species were retrieved from NCBI and extracted in FASTA format.

Multiple *ACE2* isoforms were retrieved for some species, but only one *ACE2* gene per species was selected for analysis. Many isoforms differ only in the 5′ and 3′ UTRs. For human, *ACE2* transcript variant 2 (NM_021804.3) contains 19 exons, whereas transcript variant 1 (NM_001371415.1) contains 18 exons, although both variants encode the same protein. Furthermore, some isoforms were experimentally validated, while others were predictions by automated computational approaches. We selected experimentally verified variants when available, but when only predicted variants are available, we picked one among those whose (1) amino acid identities are conserved by most other variants, and (2) sequence is not truncated (selected *ACE2* variants are listed in Supplementary File S1). Similarly, one representative coronavirus was picked out of several available strains (e.g., strain Urbani was picked for SARS-CoV).

Next, amino acid sequences of *ACE2* genes and of S protein-coding genes were aligned with MAFFT^38^ with the slow but accurate G-INS-i option. We then extracted the location and identity of amino acids that aligned to key human *ACE2* sites and to key S protein-coding gene sites in SARS-CoV-2 and SARS-CoV listed in Table 1. Mammalian *ACE2* match-mismatch heat-maps were then generated, and a total similarity score (the total number of matching amino acid identities between human and mammals at key *ACE2* sites) was calculated for each mammal. Similarly, coronavirus S protein match-mismatch heat-maps were generated (one against SARS-CoV-2 S protein and another against SARS-CoV S protein), and the total similarity score was calculated for each mammalian-specific coronavirus.

### Phylogenetic reconstruction based on 132 *ACE2* genes and 16 S protein-coding genes

Three phylogenetic trees were constructed using MAFFT G-INS-i aligned amino acid sequences with the maximum-likelihood-based PHYML approach^39^: one tree for aligned *ACE2* genes from 132 mammalian species (bootstrap = 500, model = JTT + G + I + F), another tree for *ACE2* genes from 13 sample mammalian species (bootstrap = 100, model = JTT + G + I + F), and a third tree for 16 mammalian-specific coronavirus S proteins (bootstrap = 100, model = WAG + G + I + F). All were constructed using the PHYML model implemented in DAMBE. The tree improvement option “-s” was set to “BEST” (best of NNI and SPR search). The “-o” option was set to “tlr” which optimizes the topology, branch lengths and rate parameters. Tree figure illustrations were made using the Interactive Tree Of Life (iTOL) v4^40^.

## Supplementary Information

Supplementary Information 1.

Supplementary Information 2.

## Data Availability

Supplementary file S1 contains data for mammalian *ACE2* and coronavirus S protein gene accessions, the selected species used for phylogenetic reconstruction, and key site comparisons between 132 mammalian species at aligned *ACE2* genes and between 17 coronaviruses at aligned S protein-coding genes. Supplementary file S2 contains Supplementary figures S1,S2, and S3.
