# Supplementary material for: Predicting mammalian species at risk of being infected by SARS-CoV-2 from an ACE2 perspective
Source: Sci Rep. 2021 Jan 18;11:1702. doi: 10.1038/s41598-020-80573-x (PMC7814088; doi:10.1038/s41598-020-80573-x)
Supplement: Supplementary file 1 — Supplementary Information 1. [file 41598_2020_80573_MOESM1_ESM.pdf]

Predicting mammalian species at risk of being infected by SARS-CoV-2 from an ACE2 perspective

Yulong Wei[1], Parisa Aris[1], Heba Farookhi[1], Xuhua Xia[1,2,*]

1. Department of Biology, University of Ottawa, 30 Marie Curie, P.O. Box 450, Station A, Ottawa, Ontario, Canada, K1N 6N5. Tel: (613) 562-5800 ext. 6886, Fax: (613) 562-5486.

2. Ottawa Institute of Systems Biology, Ottawa, Ontario, Canada K1H 8M5.

* Corresponding author E-mail: Xuhua.Xia@uottawa.ca

a)

Order	Family	Species	ACE2 binding hotspots similarities with hACE2															Total Similarity
Primates	*Aotidae*	*Aotus nancymaae*	S19	Q24	D30	K31	H34	E35	E37	D38	H41	E42	L79	T82	Y83	K353	R393	12/15
	Cebidae	*Sapajus apella*	S19	Q24	D30	K31	H34	E35	E37	D38	H41	E42	L79	T82	Y83	K353	R393	12/15
	Cercopithecidae	*Macaca fascicularis*	S19	Q24	D30	K31	H34	E35	E37	D38	Y41	Q42	L79	M82	Y83	K353	R393	15/15
		Macaca mulatta	S19	Q24	D30	K31	H34	E35	E37	D38	Y41	Q42	L79	M82	Y83	K353	R393	15/15
		Macaca nemestrina	S19	Q24	D30	K31	H34	E35	E37	D38	Y41	Q42	L79	M82	Y83	K353	R393	15/15
		Mandrillus leucophaeus	S19	Q24	D30	K31	H34	E35	E37	D38	Y41	Q42	L79	M82	Y83	K353	R393	15/15
		Papio anubis	S19	Q24	D30	K31	H34	E35	E37	D38	Y41	Q42	L79	M82	Y83	K353	R393	15/15
		Cercocebus atys	S19	Q24	D30	K31	H34	E35	E37	D38	Y41	Q42	L79	M82	Y83	K353	R393	15/15
		Cercopithecus aethiops	S19	Q24	D30	K31	H34	E35	E37	D38	Y41	Q42	L79	M82	Y83	K353	R393	15/15
		Chlorocebus sabaeus	S19	Q24	D30	K31	H34	E35	E37	D38	Y41	Q42	L79	M82	Y83	K353	R393	15/15
		Piliocolobus tephrosceles	S19	Q24	D30	K31	H34	E35	E37	D38	Y41	Q42	L79	M82	Y83	K353	R393	15/15
		Theropithecus gelada	S19	Q24	D30	K31	H34	E35	E37	D38	Y41	Q42	L79	M82	Y83	K353	R393	15/15
		Trachypithecus francoisi	S19	R24	D30	K31	H34	E35	E37	D38	Y41	Q42	L79	M82	Y83	K353	R393	14/15
		Rhinopithecus roxellana	S19	Q24	D30	K31	H34	E35	E37	D38	Y41	Q42	L79	M82	Y83	K353	R393	15/15
	Cheirogaleidae	*Microcebus murinus*	S45	Q50	E56	N57	N60	E61	E63	D64	H67	Q68	L105	T108	Y109	K379	R419	10/15
	Galagidae	*Otolemur garnettii*	S19	Q24	D30	N31	R34	E35	E37	E38	H41	Q42	I79	T82	Y83	K353	R393	9/15
	Callitrichidae	*Callithrix jacchus*	S19	Q24	D30	K31	H34	E35	E37	D38	H41	E42	L79	T82	Y83	K353	R393	12/15
	Cebidae	*Cebus capucinus*	S19	Q24	D30	K31	H34	E35	E37	D38	H41	E42	L79	T82	Y83	K353	R393	12/15
		Homo sapiens	S19	Q24	D30	K31	H34	E35	E37	D38	Y41	Q42	L79	M82	Y83	K353	R393	
	Hominidae	*Pan paniscus*	S19	Q24	D30	K31	H34	E35	E37	D38	Y41	Q42	L79	M82	Y83	K353	R393	15/15
		Pan troglodytes	S19	Q24	D30	K31	H34	E35	E37	D38	Y41	Q42	L79	M82	Y83	K353	R393	15/15
		Pongo abelii	S19	Q24	D30	K31	H34	E35	E37	D38	Y41	Q42	L79	M82	Y83	K353	R393	15/15
	Hylobatidae	*Hylobates moloch*	S19	Q24	D30	K31	H34	E35	E37	D38	Y41	Q42	L79	M82	Y83	K353	R393	15/15
		Nomascus leucogenys	S19	Q24	D30	K31	H34	E35	E37	D38	Y41	Q42	L79	M82	Y83	K353	R393	15/15
	Indriidae	*Propithecus coquereli*	S40	Q45	D51	K52	H55	E56	E58	D59	Y62	Q63	L100	T103	Y104	K374	R414	14/15
	Tarsiidae	*Carlito syrichta*	S19	Q24	D30	K31	Q34	E35	E37	D38	H41	Q42	I79	S82	Y83	N353	R393	10/15

Total Similarity
5 or less
6 to 7
8 to 9
10 to 11
12 to 13
14 to 15

b)

Order	Family	Species	ACE2 binding hotspots similarities with hACE2															Total Similarity
Artiodactyla	*Balaenopteridae*	*Balaenoptera acutorostrata*	S19	Q24	Q30	K31	H34	E35	E37	D38	Y41	R42	I79	T82	Y83	K352	R392	11/15
	Bovidae	*Bos indicus*	S19	Q24	E30	K31	H34	E35	E37	D38	Y41	Q42	M79	T82	Y83	K352	R392	12/15
		Bos mutus	S19	Q24	E30	K31	H34	E35	E37	D38	Y41	Q42	M79	T82	Y83	K352	R392	12/15
		Bos taurus	S19	Q24	E30	K31	H34	E35	E37	D38	Y41	Q42	M79	T82	Y83	K352	R392	12/15
		Bubalus bubalis	S18	Q23	E29	K30	H33	E34	E36	D37	Y40	Q41	M78	T81	Y82	K351	R391	12/15
		Capra hircus	S19	Q24	E30	K31	H34	E35	E37	D38	Y41	Q42	M79	T82	Y83	K352	R392	12/15
		Ovis aries	S19	Q24	E30	K31	H34	E35	E37	D38	Y41	Q42	M79	T82	Y83	K352	R392	12/15
	Camelidae	*Camelus ferus*	S19	L24	E30	E31	H34	E35	E37	D38	Y41	Q42	T79	T82	Y83	K353	R393	10/15
		Vicugna pacos	S19	L24	K30	E31	H34	E35	E37	D38	Y41	Q42	A79	I82	Y83	K353	R393	10/15
	Delphinidae	*Globicephala melas*	S19	R24	Q30	K31	R34	E35	E37	D38	Y41	Q42	I79	T82	Y83	K352	R392	10/15
		Lagenorhynchus obliquidens	S19	R24	Q30	K31	R34	E35	E37	D38	Y41	Q42	I79	T82	Y83	K352	R392	10/15
		Orcinus orca	S19	R24	Q30	K31	R34	E35	E37	D38	Y41	Q42	I79	T82	Y83	K352	R392	10/15
		Tursiops truncatus	S19	R24	Q30	K31	R34	E35	E37	D38	Y41	Q42	I79	T82	Y83	K352	R392	10/15
	Lipotidae	*Lipotes vexillifer*	S19	R24	Q30	K31	H34	E35	E37	D38	Y41	Q42	I79	T82	F83	K352	R392	10/15
	Monodontidae	*Delphinapterus leucas*	S19	Q24	Q30	K31	H34	E35	E37	D38	Y41	Q42	I79	T82	Y83	K352	R392	12/15
		Monodon monoceros	S19	Q24	Q30	K31	H34	E35	E37	D38	Y41	Q42	I79	T82	Y83	K352	R392	12/15
	Phocoenidae	*Neophocaena asiaeorientalis*	S19	Q24	Q30	K31	H34	E35	E37	D38	Y41	Q42	I79	T82	Y83	K352	R392	12/15
		Phocoena sinus	S19	Q24	Q30	K31	H34	E35	E37	D38	Y41	Q42	I79	T82	Y83	K352	R392	12/15
	Physeteridae	*Physeter catodon*	S19	Q24	Q30	K31	H34	E35	E37	D38	Y41	Q42	T79	T82	Y83	K352	R392	12/15
	Suidae	*Sus scrofa*	S19	L24	E30	K31	L34	E35	E37	D38	Y41	Q42	I79	T82	Y83	K353	R393	10/15

Total Similarity
5 or less
6 to 7
8 to 9
10 to 11
12 to 13
14 to 15

c)

Order	Family	Species	ACE2 binding hotspots similarities with hACE2															Total Similarity
Chiroptera	*Hipposideridae*	*Hipposideros armiger*	S19	L24	D30	K31	T34	E35	E37	D38	H41	L42	R79	D82	Y83	K353	R393	9/15
	Miniopteridae	*Miniopterus natalensis*	S19	K24	E30	G31	S34	Q35	E37	D38	F41	E42	L79	I82	Y83	K353	R393	7/15
	Phyllostomidae	*Phyllostomus discolor*	T19	D24	E30	N31	N34	E35	E37	E38	Y41	Q42	L79	N82	Y83	K350	R390	8/15
	Pteropodidae	*Pteropus alecto*	S19	L24	E30	K31	T34	E35	E37	D38	Y41	Q42	L79	A82	Y83	K353	K393	10/15
		Pteropus vampyrus	S19	L24	E30	K31	T34	E35	E37	D38	Y41	Q42	L79	A82	Y83	K352	K392	10/15
		Rousettus aegyptiacus	S19	L24	E30	K31	T34	E35	E37	D38	Y41	Q42	L79	T82	Y83	K353	R393	11/15
		Rousettus leschenaulti	S19	L24	E30	K31	T34	E35	E37	D38	Y41	Q42	L79	T82	Y83	K353	R393	11/15
	Rhinolophidae	*Rhinolophus ferrumequinum*	S19	L24	D30	D31	S34	E35	E37	N38	H41	Q42	L79	N82	F83	K353	R393	8/15
		Rhinolophus macrotis	S19	E24	D30	K31	S34	K35	E37	D38	Y41	E42	L79	N82	Y83	K353	R393	10/15
		Rhinolophus pusillus	S19	K24	N30	D31	S34	E35	E37	D38	Y41	Q42	I79	N82	Y83	K353	R393	9/15
		Rhinolophus sinicus	S19	R24	D30	E31	S34	E35	E37	N38	Y41	Q42	L79	N82	Y83	K353	R393	10/15
	Vespertilionidae	*Eptesicus fuscus*	S19	N24	E30	N31	S34	E35	E37	D38	H41	E42	L79	T82	Y83	K352	R392	8/15
		Myotis brandtii	S19	K24	E30	N31	S34	K35	E37	D38	H41	E42	L79	T82	Y83	K352	R392	7/15
		Myotis davidii	S19	K24	D30	N31	S34	K35	E37	D38	H41	E42	L79	T82	Y83	K352	R392	8/15
		Myotis lucifugus	S19	K24	E30	N31	S34	K35	E37	D38	H41	E42	L79	T82	Y83	K352	R392	8/15
		Pipistrellus abramus	Y19	E24	V30	K31	H34	E35	E37	N38	H41	E42	I79	G82	F83	K352	R392	6/15

Total Similarity
5 or less
6 to 7
8 to 9
10 to 11
12 to 13
14 to 15

d)

Order	Family	Species	ACE2 binding hotspots similarities with hACE2															Total Similarity
Carnivora	*Canidae*	*Canis lupus dingo*	S19	L23	E29	K30	Y33	E34	E36	E37	Y40	Q41	L78	T81	Y82	K352	R392	10/15
		Canis lupus familiaris	S19	L23	E29	K30	Y33	E34	E36	E37	Y40	Q41	L78	T81	Y82	K352	R392	10/15
		Nyctereutes procyonoides	S19	L23	E29	K30	Y33	E34	E36	E37	Y40	Q41	L78	T81	Y82	R352	R392	9/15
		Vulpes vulpes	S19	L23	E29	K30	Y33	E34	E36	E37	Y40	Q41	L78	T81	Y82	K352	R392	10/15
	Felidae	*Felis catus*	S19	L24	E30	K31	H34	E35	E37	E38	Y41	Q42	L79	T82	Y83	K353	R393	11/15
		Lynx canadensis	S19	L24	E30	K31	H34	E35	E37	E38	Y41	Q42	L79	T82	Y83	K353	R393	11/15
		Panthera tigris altaica	S11	L16	E22	K23	H26	E27	E29	E30	Y33	Q34	L71	T74	Y75	K345	R385	11/15
		Puma concolor	S19	L24	E30	K31	H34	E35	E37	E38	Y41	Q42	L79	T82	Y83	K353	R393	11/15
	Herpestidae	*Suricata suricatta*	S19	L24	E30	Q31	H34	E35	Q37	E38	Y41	L42	R79	A82	Y83	K353	R393	7/15
	Mustelidae	*Lontra canadensis*	S19	L24	E30	K31	Y34	E35	E37	E38	Y41	Q42	N79	T82	Y83	K353	R393	9/15
		Enhydra lutris kenyoni	S19	P24	E30	K31	Y34	E35	E37	E38	Y41	Q42	H79	T82	Y83	K353	R393	9/15
		Mustela erminea	S19	L24	E30	K31	Y34	E35	E37	E38	Y41	Q42	H79	T82	Y83	K353	R393	9/15
		Mustela putorius furo	S19	L24	E30	K31	Y34	E35	E37	E38	Y41	Q42	H79	T82	Y83	K353	R393	9/15
	Odobenidae	*Odobenus rosmarus*	S19	L24	E30	K31	Y34	E35	E37	E38	Y41	Q42	Q79	T82	Y83	K353	R393	9/15
	Otariidae	*Callorhinus ursinus*	S19	L24	E30	K31	S34	E35	E37	E38	Y41	Q42	Q79	T82	Y83	K353	R393	9/15
		Eumetopias jubatus	S19	L24	E30	K31	S34	E35	E37	E38	Y41	Q42	Q79	T82	Y83	K353	R393	9/15
		Zalophus californianus	S19	L24	E30	K31	S34	E35	E37	E38	Y41	Q42	Q79	T82	Y83	K353	R393	9/15
	Phocidae	*Neomonachus schauinslandi*	S19	L24	E30	K31	Y34	E35	E37	E38	Y41	Q42	Q79	T82	Y83	K353	R393	9/15
		Phoca vitulina	S19	L24	E30	K31	Y34	E35	E37	E38	Y41	Q42	Q79	T82	Y83	K353	R393	9/15
	Procyonidae	*Procyon lotor*	S19	L24	E30	N31	N34	E35	E37	E38	Y41	Q42	Q79	T82	Y83	K353	R393	8/15
	Ursidae	*Ailuropoda melanoleuca*	S19	L24	E30	K31	Y34	E35	E37	D38	Y41	Q42	H79	T82	Y83	K353	R393	10/15
		Ursus arctos	S19	L24	E30	K31	Y34	E35	E37	D38	Y41	Q42	H79	T82	Y83	K353	R393	10/15
		Ursus maritimus	S4S	L9L	E15	K16	Y19	E20	E22	D23	Y26	Q27	H64	T67	Y68	K338	R378	10/15
	Viverridae	*Paguma larvata*	S19	L24	E30	T31	Y34	E35	Q37	E38	Y41	Q42	L79	T82	Y83	K353	R393	8/15

Total Similarity
5 or less
6 to 7
8 to 9
10 to 11
12 to 13
14 to 15

e)

Order	Family	Species	ACE2 binding hotspots similarities with hACE2															Total Similarity
Rodentia	*Bathyergidae*	*Fukomys damarensis*	L19	Q24	D30	K31	Q34	E35	E37	D38	Y41	Q42	L79	A82	Y83	K353	R393	12/15
	Caviidae	*Cavia porcellus*	F27	Q32	D38	E39	L42	K43	E45	D46	Y49	Q50	L87	A90	Y91	K361	R401	10/15
	Chinchillidae	*Chinchilla lanigera*	S19	Q24	D30	N31	E34	K35	E37	D38	Y41	Q42	L79	A82	Y83	K353	R393	11/15
	Cricetidae	*Cricetulus griseus*	S19	Q24	D30	K31	Q34	E35	E37	D38	Y41	Q42	L79	N82	Y83	K353	R393	13/15
		Mesocricetus auratus	S19	Q24	D30	K31	Q34	E35	E37	D38	Y41	Q42	L79	N82	Y83	K353	R393	13/15
		Peromyscus leucopus	S19	Q24	D30	K31	Q34	E35	E37	D38	Y41	Q42	L79	N82	Y83	K353	R393	13/15
		Peromyscus maniculatus bairdii	S19	Q24	D30	K31	Q34	E35	E37	D38	Y41	Q42	L79	N82	Y83	K353	R393	13/15
		Microtus ochrogaster	S19	D24	D30	K31	Q34	E35	E37	D38	Y41	Q42	L79	S82	Y83	K353	R393	12/15
		Phodopus campbelli	S19	Q24	D30	K31	Q34	E35	E37	D38	Y41	Q42	L79	N82	Y83	K353	R393	13/15
	Dipodidae	*Jaculus jaculus*	S19	M24	D30	K31	Q34	E35	E37	D38	Y41	Q42	V79	T82	Y83	K353	R393	11/15
	Heterocephalidae	*Heterocephalus glaber*	L19	Q24	D30	K31	Q34	E35	E37	D38	Y41	Q42	L79	A82	Y83	K353	R393	12/15
	Heteromyidae	*Dipodomys ordii*	S19	L24	D30	N31	Q34	E35	E37	D38	Y41	Q42	L79	I82	Y83	K353	R393	11/15
	Muridae	*Grammomys surdaster*	S19	E24	D30	K31	Q34	E35	E37	D38	Y41	Q42	T79	N82	F83	H353	R393	9/15
		Mastomys coucha	S20	N25	N31	K32	Q35	E36	E38	D39	Y42	Q43	I80	N83	F84	H354	R394	8/15
		Mus caroli	S19	N24	N30	K31	Q34	E35	E37	D38	Y41	Q42	T79	S82	F83	H353	R393	8/15
		Mus musculus	S19	N24	N30	N31	Q34	E35	E37	D38	Y41	Q42	T79	S82	F83	H353	R393	7/15
		Mus pahari	S19	N24	N30	K31	Q34	E35	E37	D38	Y41	Q42	T79	N82	F83	H353	R393	8/15
		Rattus norvegicus	S19	K24	N30	K31	Q34	E35	E37	D38	Y41	Q42	I79	N82	F83	H353	R393	8/15
		Rattus rattus	S19	K24	N30	K31	Q34	E35	E37	D38	Y41	Q42	I79	N82	F83	H353	R393	8/15
	Octodontidae	*Octodon degus*	F45	Q50	D56	N57	Q60	K61	E63	D64	Y67	Q68	L105	A108	Y109	K379	R419	10/15
	Sciuridae	*Ictidomys tridecemlineatus*	S29	L34	D40	K41	Q44	E45	E47	D48	Y51	Q52	L89	A92	Y93	K363	R403	12/15
		Marmota flaviventris	S29	L34	D40	K41	Q44	E45	E47	D48	Y51	Q52	L89	A92	Y93	K363	R403	12/15
		Urocitellus parryii	S29	L34	D40	K41	Q44	E45	E47	D48	H51	Q52	L89	D92	Y93	K363	R403	11/15
	Spalacidae	*Nannospalax galili*	S19	Q23	D29	K30	Q33	E34	E36	D37	Y40	Q41	L78	K81	Y82	K352	R392	13/15

Total Similarity
5 or less
6 to 7
8 to 9
10 to 11
12 to 13
14 to 15

f)

Order	Family	Species	ACE2 binding hotspots similarities with hACE2															Total Similarity
Afrosoricida	*Chysochloridae*	*Chrysochloris asiatica*	S19	L23	N29	N30	Q33	E34	E36	N37	H40	Q41	L78	K81	F82	K347	R387	7/15
	Tenrecidae	*Echinops telfairi*	S19	Q24	T30	T31	N34	E35	E37	N38	Y41	Q42	L79	K82	F83	L348	R388	8/15
Cingulata	*Dasypodidae*	*Dasypus novemcinctus*	S19	Q23	E29	T30	Q33	Q34	E36	E37	H40	Q41	M78	N81	F82	K352	R392	6/15
Dasyuromorphia	*Dasyuridae*	*Sarcophilus harrisii*	L19	M24	E30	N31	K34	A35	E37	D38	Y41	Q42	L79	A82	Y83	K353	R393	8/15
Diprotodontia	*Phascolarctidae*	*Phascolarctos cinereus*	F19	R24	E30	T31	K34	E35	E37	E38	Y41	Q42	I79	T82	F83	K353	R393	6/15
	Vombatidae	*Vombatus ursinus*	F21	R26	E32	T33	K36	E37	E39	E40	Y43	Q44	I81	T84	F85	K355	R395	6/15
Eulipotyphla	*Erinaceidae*	*Erinaceus europaeus*	T19	E24	D30	D31	R34	Q35	E37	N38	Y41	E42	T79	N82	Y83	N353	R393	5/15
	Talpidae	*Condylura cristata*	S19	Q24	E30	T31	R34	E35	E37	E38	Y41	N42	D79	R82	F83	K348	R388	7/15
	Soricidae	*Sorex araneus*	S19	N23	E29	N30	K33	D34	E36	D37	Y40	N41	I78	T81	F82	K351	R391	6/15
Lagomorpha	*Ochotonidae*	*Ochotona princeps*	S22	L27	D33	K34	Q37	E38	E40	D41	Y44	Q45	L82	T85	Y86	K356	R396	12/15
	Leporidae	*Oryctolagus cuniculus*	S19	L24	E30	K31	Q34	E35	E37	D38	Y41	Q42	L79	T82	Y83	K353	R393	11/15
Macroscelidae	*Macroscelididae*	*Elephantulus edwardii*	P19	Q24	E30	Q31	Q34	Q35	E37	D38	Y41	Q42	V79	N82	F83	K348	R388	6/15
Monotremata	*Ornithorhynchidae*	*Ornithorhynchus anatinus*	K19	E23	T29	Q30	K33	Q34	E36	D37	Y40	Q41	N78	K81	F82	K352	R392	6/15
Perissodactyla	*Equidae*	*Equus asinus*	S19	L24	E30	K31	S34	E35	E37	E38	H41	Q42	L79	T82	Y83	K331	R371	9/15
		Equus caballus	S19	L24	E30	K31	S34	E35	E37	E38	H41	Q42	L79	T82	Y83	K353	R393	9/15
		Equus przewalskii	S19	L24	E30	K31	S34	E35	E37	E38	H41	Q42	L79	T82	Y83	K353	R393	9/15
	Rhinocerotidae	*Ceratotherium simum*	S19	L24	E30	K31	P34	E35	E37	D38	Y41	Q42	L79	T82	Y83	K353	R393	11/15
Pholidota	*Manidae*	*Manis javanica*	S19	E24	E30	K31	S34	E35	E37	E38	Y41	Q42	I79	N82	Y83	K353	R393	9/15
Proboscidea	*Elephantidae*	*Loxodonta africana*	S19	L24	D30	T31	Q34	E35	E37	D38	Y41	Q42	L79	D82	F83	K348	R388	10/15
Scandentia	*Tupaiidae*	*Tupaia chinensis*	T19	E24	N30	K31	I34	E35	E37	E38	H41	Q42	Q79	R82	Y83	K353	R393	7/15
Sirenia	*Trichechidae*	*Trichechus manatus latirostris*	S19	L24	D30	T31	Q34	E35	E37	D38	Y41	Q42	L79	N82	F83	K348	R388	10/15
Tubulidentata	*Orycteropodidae*	*Orycteropus afer*	A19	L24	E30	K31	Q34	E35	E37	N38	Y41	Q42	I79	S82	F83	-	-	5/15

Total Similarity
5 or less
6 to 7
8 to 9
10 to 11
12 to 13
14 to 15

Supplementary Fig. S1

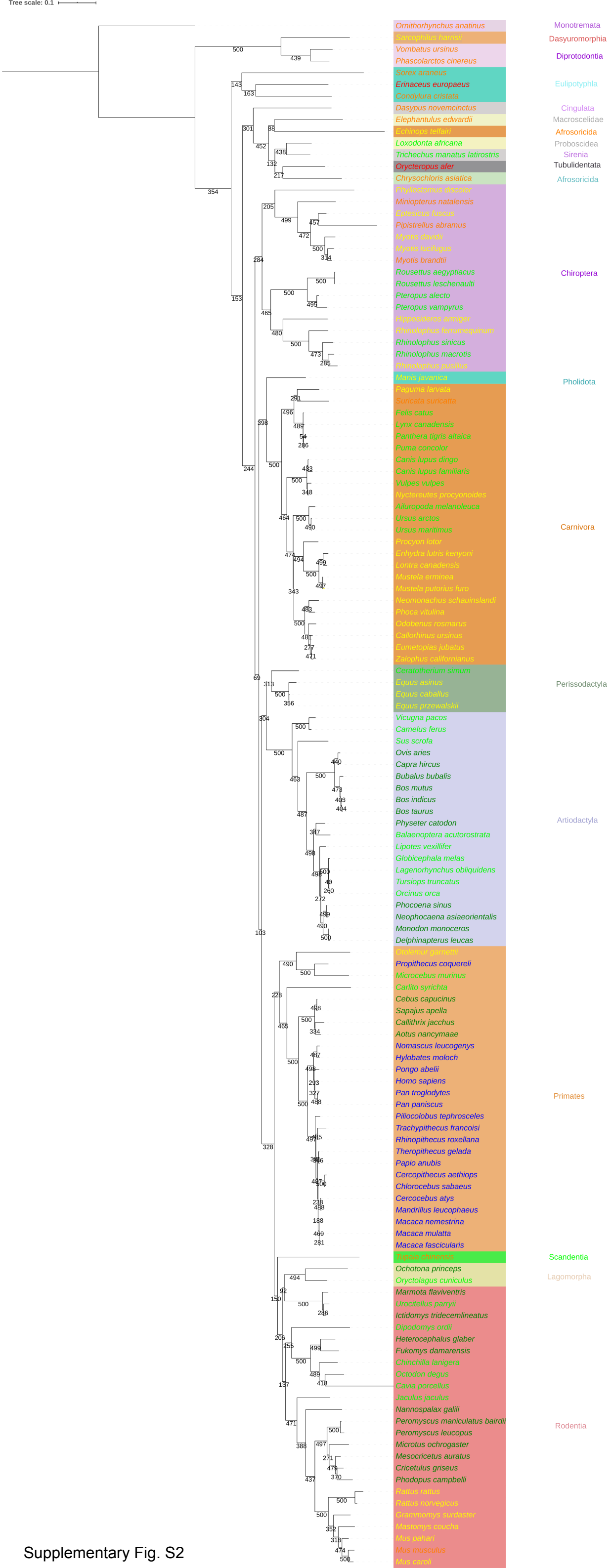

Supplementary Fig. S2

Order	Family	Species	ACE2 binding hotspots similiarties with hACE2										Total Similarity
Afrosoricida	Chysochloridae	Chrysochloris asiatica	N30	H40	K81	F82	S83	K347	D348	D349	F350	R351	4
	Tenrecidae	Echinops telfairi	T31	Y41	K82	F83	S84	L348	N349	D350	F351	R352	4
Artiodactyla	Balaenopteridae	Balaenoptera acutorostrata	K31	Y41	T82	Y83	P84	K352	G353	D354	F355	R356	9
	Bovidae	Bos indicus	K31	Y41	T82	Y83	S84	K352	G353	D354	F355	R356	8
		Bos mutus	K31	Y41	T82	Y83	S84	K352	G353	D354	F355	R356	8
		Bos taurus	K31	Y41	T82	Y83	S84	K352	G353	D354	F355	R356	8
		Bubalus bubalis	K30	Y40	T81	Y82	S83	K351	G352	D353	F354	R355	8
		Capra hircus	K31	Y41	T82	Y83	S84	K352	G353	D354	F355	R356	8
		Ovis aries	K31	Y41	T82	Y83	S84	K352	G353	D354	F355	R356	8
	Camelidae	Camelus ferus	E31	Y41	T82	Y83	P84	K353	G354	D355	F356	R357	8
		Vicugna pacos	E31	Y41	I82	Y83	P84	K353	G354	D355	F356	R357	8
	Delphinidae	Globicephala melas	K31	Y41	T82	Y83	P84	K352	G353	D354	F355	R356	9
		Lagenorhynchus obliquidens	K31	Y41	T82	Y83	P84	K352	G353	D354	F355	R356	9
		Orcinus orca	K31	Y41	T82	Y83	P84	K352	G353	D354	F355	R356	9
		Tursiops truncatus	K31	Y41	T82	Y83	P84	K352	G353	D354	F355	R356	9
	Lipotidae	Lipotes vexillifer	K31	Y41	T82	F83	P84	K352	G353	D354	F355	R356	8
	Monodontidae	Delphinapterus leucas	K31	Y41	T82	Y83	P84	K352	G353	D354	F355	R356	9
		Monodon monoceros	K31	Y41	T82	Y83	P84	K352	G353	D354	F355	R356	9
	Phocoenidae	Neophocaena asiaeorientalis	K31	Y41	T82	Y83	S84	K352	G353	D354	F355	R356	8
		Phocoena sinus	K31	Y41	T82	Y83	S84	K352	G353	D354	F355	R356	8
	Physeteridae	Physeter catodon	K31	Y41	T82	Y83	P84	K352	G353	D354	F355	R356	9
	Suidae	Sus scrofa	K31	Y41	T82	Y83	P84	K353	G354	D355	F356	R357	9
Carnivora	Canidae	Canis lupus dingo	K30	Y40	T81	Y82	P83	K352	G353	D354	F355	R356	9
		Canis lupus familiaris	K30	Y40	T81	Y82	P83	K352	G353	D354	F355	R356	9
		Nyctereutes procyonoides	K30	Y40	T81	Y82	P83	R352	G353	D354	F355	R356	8
		Vulpes vulpes	K30	Y40	T81	Y82	P83	K352	G353	D354	F355	R356	9
	Felidae	Felis catus	K31	Y41	T82	Y83	P84	K353	G354	D355	F356	R357	9
		Lynx canadensis	K31	Y41	T82	Y83	P84	K353	G354	D355	F356	R357	9
		Panthera tigris altaica	K23	Y33	T74	Y75	P76	K345	G346	D347	F348	R349	9
		Puma concolor	K31	Y41	T82	Y83	P84	K353	G354	D355	F356	R357	9
	Herpestidae	Suricata suricatta	Q31	Y41	A82	Y83	P84	K353	G354	D355	F356	R357	8
	Mustelidae	Lontra canadensis	K31	Y41	T82	Y83	P84	K353	R354	D355	F356	R357	8
		Enhydra lutris kenyoni	K31	Y41	T82	Y83	P84	K353	R354	D355	F356	R357	8
		Mustela erminea	K31	Y41	T82	Y83	P84	K353	R354	D355	F356	R357	8
		Mustela putorius furo	K31	Y41	T82	Y83	P84	K353	R354	D355	F356	R357	8
	Odobenidae	Odobenus rosmarus	K31	Y41	T82	Y83	P84	K353	H354	D355	F356	R357	8
	Otariidae	Callorhinus ursinus	K31	Y41	T82	Y83	P84	K353	H354	D355	F356	R357	8
		Eumetopias jubatus	K31	Y41	T82	Y83	P84	K353	H354	D355	F356	R357	8
		Zalophus californianus	K31	Y41	T82	Y83	P84	K353	H354	D355	F356	R357	8
	Phocidae	Neomonachus schauinslandi	K31	Y41	T82	Y83	P84	K353	H354	D355	F356	R357	8
		Phoca vitulina	K31	Y41	T82	Y83	P84	K353	R354	D355	F356	R357	8
	Procyonidae	Procyon lotor	N31	Y41	T82	Y83	P84	K353	G354	D355	F356	R357	8
	Ursidae	Ailuropoda melanoleuca	K31	Y41	T82	Y83	P84	K353	G354	D355	F356	R357	9
		Ursus arctos	K31	Y41	T82	Y83	P84	K353	G354	D355	F356	R357	9
		Ursus maritimus	K16	Y26	T67	Y68	P69	K338	G339	D340	F341	R342	9
	Viverridae	Paguma larvata	T31	Y41	T82	Y83	P84	K353	G354	D355	F356	R357	8
Chiroptera	Hipposideridae	Hipposideros armiger	K31	H41	D82	Y83	R84	K353	G354	D355	F356	R357	7
	Miniopteridae	Miniopterus natalensis	G31	F41	I82	Y83	P84	K353	G354	D355	F356	R357	7
	Phyllostomidae	Phyllostomus discolor	N31	Y41	N82	Y83	P84	K350	K351	D352	F353	R354	7
	Pteropodidae	Pteropus alecto	K31	Y41	A82	Y83	Q84	K353	G354	D355	F356	R357	8
		Pteropus vampyrus	K31	Y41	A82	Y83	Q84	K352	G353	D354	F355	R356	8
		Rousettus aegyptiacus	K31	Y41	T82	Y83	Q84	K353	G354	D355	F356	R357	8
		Rousettus leschenaulti	K31	Y41	T82	Y83	Q84	K353	G354	D355	F356	R357	8
	Rhinolophidae	Rhinolophus ferrumequinum	D31	H41	N82	F83	S84	K353	G354	D355	F356	R357	5
		Rhinolophus macrotis	K31	Y41	N82	Y83	P84	K353	G354	D355	F356	R357	9
		Rhinolophus pusillus	D31	Y41	N82	Y83	P84	K353	G354	D355	F356	R357	8
		Rhinolophus sinicus	E31	Y41	N82	Y83	P84	K353	G354	D355	F356	R357	8
	Vespertilionidae	Eptesicus fuscus	N31	H41	T82	Y83	P84	K352	N353	D354	F355	R356	6
		Myotis brandtii	N31	H41	T82	Y83	P84	K352	G353	D354	F355	R356	7
		Myotis davidii	N31	H41	T82	Y83	P84	K352	G353	D354	F355	R356	7
		Myotis lucifugus	N31	H41	T82	Y83	P84	K352	G353	D354	F355	R356	7
		Pipistrellus abramus	K31	H41	G82	F83	P84	K352	N353	D354	F355	R356	6
Cingulata	Dasypodidae	Dasypus novemcinctus	T30	H40	N81	F82	S83	K352	G353	D354	F355	R356	5
Dasyuromorphia	Dasyuridae	Sarcophilus harrisii	N31	Y41	A82	Y83	P84	K353	G354	D355	F356	R357	8
Diprotodontia	Phascolarctidae	Phascolarctos cinereus	T31	Y41	T82	F83	P84	K353	G354	D355	F356	R357	7
	Vombatidae	Vombatus ursinus	T33	Y43	T84	F85	P86	K355	G356	D357	F358	R359	7
Eulipotyphla	Erinaceidae	Erinaceus europaeus	D31	Y41	N82	Y83	P84	N353	G354	D355	F356	R357	7
	Talpidae	Condylura cristata	T31	Y41	R82	F83	Q84	K348	G349	D350	F351	R352	6
	Soricidae	Sorex araneus	N30	Y40	T81	F82	P83	K351	N352	D353	Y354	R355	5
Lagomorpha	Ochotonidae	Ochotona princeps	K34	Y44	T85	Y86	P87	K356	D357	D358	F359	R360	8
	Leporidae	Oryctolagus cuniculus	K31	Y41	T82	Y83	P84	K353	R354	D355	F356	R357	8
Macroscelidae	Macroscelididae	Elephantulus edwardii	Q31	Y41	N82	F83	P84	K348	G349	D350	F351	R352	7
Monotremata	Ornithorhynchidae	Ornithorhynchus anatinus	Q30	Y40	K81	F82	N83	K352	N353	D354	F355	R356	5
Perissodactyla	Equidae	Equus asinus	K31	H41	T82	Y83	P84	K331	G332	D333	F334	R335	8
		Equus caballus	K31	H41	T82	Y83	P84	K353	G354	D355	F356	R357	8
		Equus przewalskii	K31	H41	T82	Y83	P84	K353	G354	D355	F356	R357	8
	Rhinocerotidae	Ceratotherium simum	K31	Y41	T82	Y83	P84	K353	G354	D355	F356	R357	9
Pholidota	Manidae	Manis javanica	K31	Y41	N82	Y83	Q84	K353	H354	D355	F356	R357	7
Primates	Aotidae	Aotus nancymaae	K31	H41	T82	Y83	P84	K353	Q354	D355	F356	R357	6
	Cebidae	Sapajus apella	K31	H41	T82	Y83	P84	K353	Q354	D355	F356	R357	7
	Cercopithecidae	Macaca fascicularis	K31	Y41	M82	Y83	P84	K353	G354	D355	F356	R357	10
		Cercocebus atys	K31	Y41	M82	Y83	P84	K353	G354	D355	F356	R357	10
		Cercopithecus aethiops	K31	Y41	M82	Y83	P84	K353	G354	D355	F356	R357	10
		Chlorocebus sabaeus	K31	Y41	M82	Y83	P84	K353	G354	D355	F356	R357	10
		Macaca mulatta	K31	Y41	M82	Y83	P84	K353	G354	D355	F356	R357	10
		Macaca nemestrina	K31	Y41	M82	Y83	P84	K353	G354	D355	F356	R357	10
		Mandrillus leucophaeus	K31	Y41	M82	Y83	P84	K353	G354	D355	F356	R357	10
		Papio anubis	K31	Y41	M82	Y83	P84	K353	G354	D355	F356	R357	10
		Piliocolobus tephrosceles	K31	Y41	M82	Y83	P84	K353	G354	D355	F356	R357	10
		Theropithecus gelada	K31	Y41	M82	Y83	P84	K353	G354	D355	F356	R357	10
		Rhinopithecus roxellana	K31	Y41	M82	Y83	P84	K353	G354	D355	F356	R357	10
		Trachypithecus francoisi	K31	Y41	M82	Y83	P84	K353	G354	D355	F356	R357	10
	Cheirogaleidae	Microcebus murinus	N57	H67	T108	Y109	P110	K379	G380	D381	F382	R383	7
	Galagidae	Otolemur garnettii	N31	H41	T82	Y83	P84	K353	D354	D355	F356	R357	6
	Callitrichidae	Callithrix jacchus	K31	H41	T82	Y83	P84	K353	Q354	D355	F356	R357	7
	Cebidae	Cebus capucinus	K31	H41	T82	Y83	P84	K353	Q354	D355	F356	R357	7
	Hominidae	Homo sapiens	K31	Y41	M82	Y83	P84	K353	G354	D355	F356	R357	
		Pan paniscus	K31	Y41	M82	Y83	P84	K353	G354	D355	F356	R357	10
		Pan troglodytes	K31	Y41	M82	Y83	P84	K353	G354	D355	F356	R357	10
		Pongo abelii	K31	Y41	M82	Y83	P84	K353	G354	D355	F356	R357	10
	Hylobatidae	Hylobates moloch	K31	Y41	M82	Y83	P84	K353	G354	D355	F356	R357	10
		Nomascus leucogenys	K31	Y41	M82	Y83	P84	K353	G354	D355	F356	R357	10
	Indriidae	Propithecus coquereli	K52	Y62	T103	Y104	P105	K374	G375	D376	F377	R378	9
	Tarsiidae	Carlito syrichta	K31	H41	S82	Y83	P84	N353	S354	D355	F356	R357	6
Proboscidea	Elephantidae	Loxodonta africana	T31	Y41	D82	F83	P84	K348	G349	D350	F351	R352	7
Rodentia	Bathyergidae	Fukomys damarensis	K31	Y41	A82	Y83	P84	K353	N354	D355	F356	R357	8
	Caviidae	Cavia porcellus	E39	Y49	A90	Y91	P92	K361	N362	D363	F364	R365	7
	Chinchillidae	Chinchilla lanigera	N31	Y41	A82	Y83	P84	K353	D354	D355	F356	R357	7
	Cricetidae	Cricetulus griseus	K31	Y41	N82	Y83	S84	K353	G354	D355	F356	R357	8
		Microtus ochrogaster	K31	Y41	S82	Y83	S84	K353	D354	D355	F356	R357	7
		Mesocricetus auratus	K31	Y41	N82	Y83	S84	K353	G354	D355	F356	R357	8
		Peromyscus leucopus	K31	Y41	N82	Y83	S84	K353	G354	D355	F356	R357	8
		Peromyscus maniculatus bairdii	K31	Y41	N82	Y83	S84	K353	G354	D355	F356	R357	8
		Phodopus campbelli	K31	Y41	N82	Y83	P84	K353	E354	D355	F356	R357	8
	Dipodidae	Jaculus jaculus	K31	Y41	T82	Y83	P84	K353	N354	D355	F356	R357	8
	Heterocephalidae	Heterocephalus glaber	K31	Y41	A82	Y83	S84	K353	D354	D355	F356	R357	7
	Heteromyidae	Dipodomys ordii	N31	Y41	I82	Y83	S84	K353	G354	D355	F356	R357	7
	Muridae	Grammomys surdaster	K31	Y41	N82	F83	S84	H353	G354	D355	F356	R357	6
		Mastomys coucha	K32	Y42	N83	F84	S85	H354	G355	D356	F357	R358	6
		Mus caroli	K31	Y41	S82	F83	S84	H353	G354	D355	F356	R357	6
		Mus musculus	N31	Y41	S82	F83	S84	H353	G354	D355	F356	R357	5
		Mus pahari	K31	Y41	N82	F83	S84	H353	G354	D355	F356	R357	6
		Rattus norvegicus	K31	Y41	N82	F83	S84	H353	G354	D355	F356	R357	6
		Rattus rattus	K31	Y41	N82	F83	S84	H353	G354	D355	F356	R357	6
	Octodontidae	Octodon degus	N57	Y67	A108	Y109	P110	K379	N380	D381	F382	R383	7
	Sciuridae	Ictidomys tridecemlineatus	K41	Y51	A92	Y93	P94	K363	G364	D365	F366	R367	9
		Marmota flaviventris	K41	Y51	A92	Y93	P94	K363	G364	D365	F366	R367	9
		Urocitellus parryii	K41	H51	D92	Y93	P94	K363	G364	D365	F366	R367	8
	Spalacidae	Nannospalax galili	K30	Y40	K81	Y82	S83	K352	G353	D354	F355	R356	8
Scandentia	Tupaiidae	Tupaia chinensis	K31	H41	R82	Y83	P84	K353	N354	D355	F356	R357	7
Sirenia	Trichechidae	Trichechus manatus latirostris	T31	Y41	N82	F83	P84	K348	G349	D350	F351	R352	7
Tubulidentata	Orycteropodidae	Orycteropus afer	K31	Y41	S82	F83	S84	G346	K3547	G348	D349	F350	2

Total similarity
0 to 1
2 to 3
4 to 5
6 to 7
8 to 9
10

Supplementary Fig. S3

SUPPLEMENTARY FIGURE LEGENDS

Supplementary Fig. S1. Comparisons of *ACE2* genes in 132 mammalian species at 15 key human ACE2 sites involved in SARS-CoV-2 S protein binding. These species belong to the orders a) Primates, b) Artiodactyla, c) Chiroptera, d) Canivora, and e) Rodentia, and in panel f) displays species belonging to 14 other orders. All *ACE2* genes are aligned using MAFFT with option G-INS-i. Highlighted blue and red are matching and mis-matching amino acid residues, respectively, between mammalian *ACE2* and human *ACE2*. Total similarity designates the total number of matching amino acid residues with respect to human *ACE2*. The total similarity score for mammalian *ACE2* in blue highlights perfect site similarities (14 to 15 matching sites), in green highlights high similarity (12 to 13 matching sites), in light green highlights medium-high similarity (10 to 11 matching sites), in yellow highlights medium similarity (8 to 9 matching sites), in orange highlights medium-low similarity (6 to 7 matching sites), and in red highlights low similarity (5 or less matching sites).

Supplementary Fig. S2. Phylogenetic reconstruction based on 132 MAFFT G-INS-i aligned mammalian *ACE2* amino acid sequences. The tree is built with the maximum-likelihood-based PHYML approach, with best substitution model = JTT + G + I + F, Bootstrap = 500, and Outgroup = *Ornithorhynchus anatinus*. Mammalian species names are colored based on total similarity at *ACE2* against human *ACE2*: in blue highlights perfect site similarity (14 to 15 matching sites), in green highlights high similarity (12 to 13 matching sites), in light green highlights medium-high similarity (10 to 11 matching sites), in yellow highlights medium similarity (8 to 9 matching sites), in orange highlights medium-low similarity (6 to 7 matching sites), and in red highlights low similarity (5 or less matching sites).

Supplementary Fig. S3. Comparisons of *ACE2* genes in 132 mammalian species at 10 key human ACE2 sites involved in SARS-CoV S protein binding. These species belong to the orders Primates, Artiodactyla, Chiroptera, Canivora, Rodentia, and 14 other orders. All *ACE2* genes are aligned using MAFFT with option G-INS-i. Highlighted blue and red are matching and mis-matching amino acid residues, respectively, between mammalian *ACE2* and human *ACE2*. Total similarity designates the total number of matching amino acid residues with respect to human *ACE2*. The total similarity score for mammalian *ACE2* in blue highlights perfect site similarities (10 matching sites), in green highlights high similarity (8 to 9 matching sites), in light green highlights medium-high similarity (6 to 7 matching sites), in yellow highlights medium similarity (4 to 5 matching sites), in orange highlights medium-low similarity (2 to 3 matching sites), and in red highlights low similarity (1 or less matching sites).
